# Impact of Donor Volatile Anesthetic Choice on Recipient Post-Reperfusion Syndrome and Clinical Outcomes in Living Donor Liver Transplantation

**DOI:** 10.3390/ijms27083465

**Published:** 2026-04-13

**Authors:** Hyeun-Joon Bae, Shiyeun Lee, Kyoung-Sun Kim, Hye-Mee Kwon, In-Gu Jun, Jun-Gol Song, Gyu-Sam Hwang

**Affiliations:** Department of Anesthesiology and Pain Medicine, Asan Medical Center, University of Ulsan College of Medicine, 88 Olympic-ro 43-gil, Songpa-gu, Seoul 05505, Republic of Korea; joshpheonix1@gmail.com (H.-J.B.); sophist2036@naver.com (S.L.); kyoungsun.kim@amc.seoul.kr (K.-S.K.); hyemee.kwon@amc.seoul.kr (H.-M.K.); kshwang@amc.seoul.kr (G.-S.H.)

**Keywords:** living donor liver transplantation, post-reperfusion syndrome, volatile anesthetics, sevoflurane, anesthetic preconditioning

## Abstract

Post-reperfusion syndrome (PRS) remains a critical complication in living donor liver transplantation (LDLT). While sevoflurane is recognized for its organ-protective properties, the impact of the donor’s anesthetic choice on recipient outcomes has not been clearly established. This study evaluated whether donor sevoflurane anesthesia reduces the incidence of PRS in recipients compared to desflurane. We retrospectively analyzed 5006 adult LDLT recipients whose donors received either sevoflurane or desflurane. Propensity score matching was employed to minimize selection bias, resulting in 941 matched pairs. The incidence of PRS was significantly lower in the sevoflurane group (64.0%) compared to the desflurane group (71.8%; *p* < 0.001). Multivariable logistic regression identified donor sevoflurane as an independent protective factor against PRS (odds ratio 0.47; 95% confidence interval 0.41–0.55; *p* < 0.001). Furthermore, recipients in the sevoflurane group exhibited significantly lower rates of prolonged intensive care unit stay, along with superior recovery of liver enzymes and inflammatory markers. Our findings suggest a potential association between donor sevoflurane anesthesia and more favorable early recipient outcomes, including a reduced incidence of PRS and enhanced recovery.

## 1. Introduction

Liver transplantation (LT) is the established definitive treatment for patients with end-stage liver disease [1]. Despite advancements in surgical techniques and perioperative management, post-reperfusion syndrome (PRS)—characterized by severe hemodynamic instability immediately following graft reperfusion—remains a critical intraoperative complication [2,3,4]. Clinically, PRS is defined as a decrease in mean arterial pressure (MAP) of more than 30% from the baseline value, lasting at least 1 min within the first 5 min of reperfusion. PRS is associated with significant adverse clinical outcomes, including lower early survival rates, prolonged intensive care unit (ICU) stays, and extended durations of mechanical ventilation [5,6]. Although predicting and preventing PRS remains challenging due to the multifactorial nature of its pathogenesis, various pharmacological interventions have been investigated to improve clinical outcomes [3,7].

Sevoflurane has been suggested to possess organ-protective properties beyond its anesthetic effects [8,9,10]. While its protective effects against hepatic ischemia–reperfusion injury have been demonstrated in various animal studies [11,12,13], robust clinical investigations remain relatively limited. Furthermore, clinical research investigating the protective effects of these agents has primarily focused on the choice of anesthetic for the recipient [8,14]. This focus may not fully address the initial ischemic insult to the liver graft, which begins earlier during donor hepatectomy.

Sevoflurane and desflurane are widely regarded as the volatile agents of choice for living donors due to their favorable pharmacokinetic profiles [15,16]. Previous studies have demonstrated that the choice between these two agents does not affect postoperative hepatic or renal function in living donors [17,18]. In contrast, research involving transplant recipients has shown that the choice of volatile anesthetic administered to the recipient can significantly impact their clinical outcomes [19,20,21]. However, to the best of our knowledge, no study has directly compared the impact of the donor’s volatile anesthetic choice on recipient outcomes. Although deceased donor liver transplantation remains the predominant procedure in Western countries, the annual volume of living donor liver transplantation (LDLT) has steadily increased over the past decade [22]. Despite comprising a minority of all liver transplants, LDLT has demonstrated superior clinical outcomes, highlighting the importance of clinical research on LDLT to optimize perioperative management [23]. Therefore, this study aimed to evaluate the impact of the donor’s volatile anesthetic choice (sevoflurane vs. desflurane) on the incidence of PRS and postoperative outcomes in a large cohort of adult LDLT recipients.

## 2. Results

### 2.1. Study Population and Propensity Score Matching Analysis

**Figure 1:**
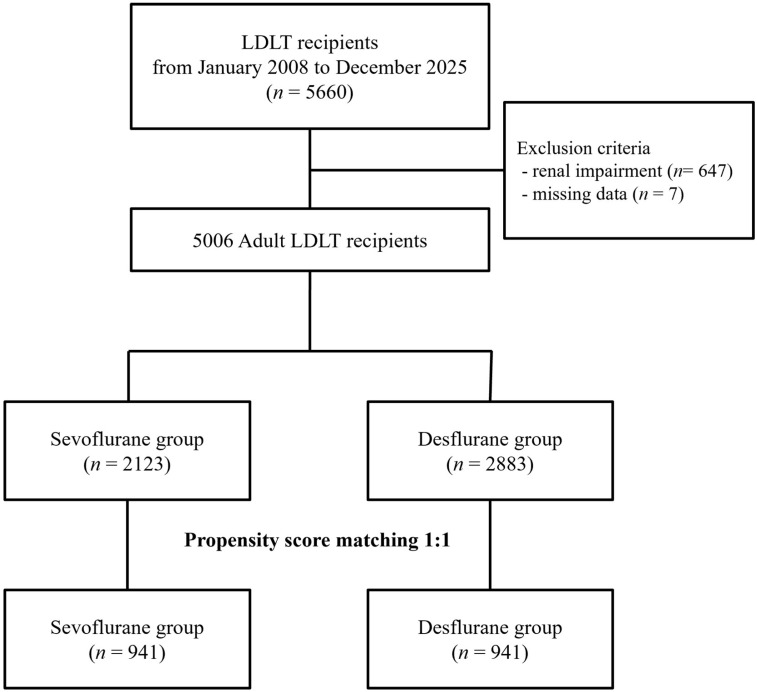
Flowchart of the study population. Abbreviations: LDLT, living donor liver transplantation.

A total of 5006 adult recipients who underwent primary LDLT during the study period were included in the final analysis, consisting of 2123 (42.4%) in the sevoflurane group and 2883 (57.6%) in the desflurane group based on the volatile anesthetic administered to the donor (Figure 1). Before propensity score matching (PSM), differences in baseline characteristics were observed between the two groups (Table 1). After 1:1 PSM, 941 pairs of recipients (total *n* = 1882) were generated, successfully balancing all baseline covariates. Following matching, all covariates exhibited standardized mean differences of less than 0.1, indicating a well-balanced cohort across demographic, preoperative, and intraoperative variables.

### 2.2. Primary Outcome

In the unadjusted cohort, the incidence of PRS was significantly lower in the sevoflurane group (51.5% vs. 80.4%; odds ratio [OR] 0.26, 95% confidence interval [CI] 0.23–0.29, *p* < 0.001). Multivariable analysis confirmed donor sevoflurane as an independent protective factor against PRS (adjusted OR 0.47, 95% CI 0.41–0.55, *p* < 0.001; Table 2). This result was consistent in the matched cohort (64.0% vs. 71.8%; OR 0.70, 95% CI 0.57–0.85, *p* < 0.001).

### 2.3. Secondary Outcomes

Similarly to the primary outcome, postoperative clinical outcomes were evaluated across the three analytical models (Table 3). In the unadjusted cohort, the incidence of prolonged ICU stay (>30 days) was significantly lower in the sevoflurane group compared to the desflurane group (1.4% vs. 3.5%; OR 0.40, 95% CI 0.26–0.59, *p* < 0.001). Multivariable analysis confirmed this protective effect (adjusted OR 0.49, 95% CI 0.30–0.79, *p* = 0.004). This result remained consistent within the matched cohort (1.3% vs. 2.8%; OR 0.45, 95% CI 0.22–0.89, *p* = 0.025), where the median ICU length of stay was also statistically shorter in the sevoflurane group (3.0 vs. 3.0 days, *p* = 0.039). For prolonged mechanical ventilation (>14 days), the incidence was also significantly lower in the sevoflurane group in both the unadjusted (2.2% vs. 4.6%; OR 0.47, 95% CI 0.33–0.65, *p* < 0.001) and multivariable-adjusted models (adjusted OR 0.60, 95% CI 0.41–0.88, *p* = 0.011). However, within the matched cohort, this difference was no longer statistically significant (2.6% vs. 3.9%; OR 0.64, 95% CI 0.37–1.07, *p* = 0.093), and the median duration of mechanical ventilation was similar between the groups (2.0 vs. 2.0 days, *p* = 0.880). Regarding other secondary outcomes in the matched cohort, the incidences of early allograft dysfunction (7.1% vs. 8.2%, *p* = 0.435) and 1-year graft failure (5.1% vs. 4.3%, *p* = 0.445) did not differ significantly between the two groups.

**Figure 2:**
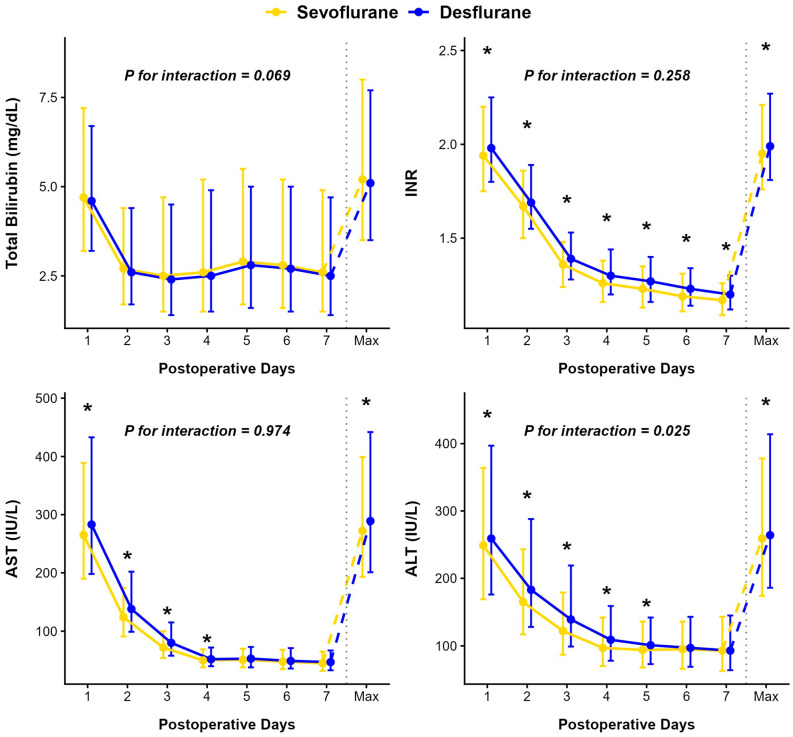
Comparison of postoperative trends between the Sevoflurane (yellow) and Desflurane (blue) groups. The data are presented as medians with error bars representing the interquartile range. The solid lines indicate the trend from postoperative day 1 to 7 and maximum values are shown to the right of the dashed line. Asterisks (*) indicate a statistically significant difference (*p* < 0.05) between the two groups at each time point. P for interaction values are derived from the Linear Quantile Mixed Model to evaluate the differences in changes over time between the groups. Abbreviations: INR, international normalized ratio; AST, aspartate aminotransferase; ALT, alanine aminotransferase.

**Figure 3:**
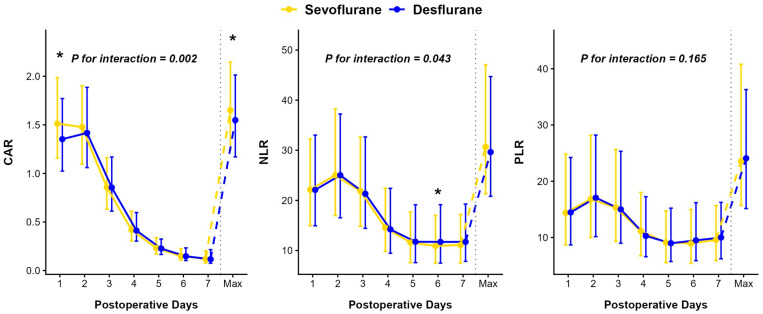
Comparison of postoperative trends between the Sevoflurane (yellow) and Desflurane (blue) groups. The data are presented as medians with error bars representing the interquartile range. The solid lines indicate the trend from postoperative day 1 to 7 and maximum values are shown to the right of the dashed line. Asterisks (*) indicate a statistically significant difference (*p* < 0.05) between the two groups at each time point. *p*-Values are derived from the Linear Quantile Mixed Model to evaluate the differences in changes over time between the groups. Abbreviations: CAR, C-reactive protein-to-albumin ratio; NLR, neutrophil-to-lymphocyte ratio; PLR, platelet-to-lymphocyte ratio.

The median peak values of prothrombin time-international normalized ratio (INR), aspartate aminotransferase (AST), and alanine aminotransferase (ALT) were significantly lower in the sevoflurane group compared to the desflurane group, whereas no significant difference was observed for total bilirubin (TB) (Table 4). Although the overall longitudinal trends for AST (*p* = 0.974), INR (*p* = 0.258), and total bilirubin (*p* = 0.069) did not differ significantly between the groups, cross-sectional analyses at specific time points revealed significant daily differences. Specifically, the sevoflurane group exhibited significantly lower AST levels from postoperative days 1 to 4, and consistently lower INR levels throughout the entire 7-day postoperative period compared to the desflurane group (Figure 2). Regarding inflammatory markers, a significant group-by-time interaction was observed for both the C-reactive protein-to-albumin ratio (CAR, *p* = 0.002) and neutrophil-to-lymphocyte ratio (NLR, *p* = 0.043), reflecting more favorable recovery trajectories in the sevoflurane group (Figure 3). Conversely, the interaction between time and group for the platelet-to-lymphocyte ratio (PLR) was not statistically significant (*p* = 0.165).

## 3. Discussion

Our results demonstrated that the use of sevoflurane in living donors was significantly associated with a reduced incidence of PRS in recipients. Furthermore, recipients in the sevoflurane group exhibited better postoperative clinical outcomes, including lower rates of prolonged ICU stay. The sevoflurane group showed significantly lower peak levels of INR, AST, and ALT, alongside more favorable recovery trends in inflammatory indices (CAR, NLR) during the first postoperative week.

Although the fundamental pathophysiology of PRS is multifactorial and not yet fully understood, ischemia–reperfusion injury (IRI) is widely recognized as playing a pivotal role [4,7]. During the ischemic phase, cellular hypoxia and metabolic distress lead to the accumulation of toxic metabolites, reactive oxygen species (ROS), and proinflammatory cytokines within the liver graft [24]. Upon reperfusion, these accumulated mediators are abruptly released into the systemic circulation, triggering the profound vasodilation and myocardial depression characteristic of PRS [24,25]. Therefore, strategies to mitigate the initial IRI are essential for preventing these adverse hemodynamic events.

The organ-protective potential of volatile anesthetics has been investigated under the concept of anesthetic preconditioning (APC) [8,26]. Experimental studies have demonstrated that sevoflurane preconditioning effectively attenuates hepatic ischemia–reperfusion injury and facilitates hemodynamic recovery by suppressing inflammatory responses and oxidative stress [27,28]. These protective effects have also been observed in clinical settings involving major liver resection. A randomized controlled trial showed that pharmacological preconditioning with sevoflurane significantly reduced postoperative liver injury and decreased the overall rate of complications compared to propofol-based anesthesia [29]. Desflurane has also been reported to modulate the inflammatory response during hepatectomy by regulating the expression of matrix metalloproteinases, which are key markers of tissue remodeling and inflammation [30]. Collectively, these findings support the possibility that volatile agents possess properties that help precondition the liver against ischemic stress.

Clinical studies involving liver transplant recipients have indicated that the choice of volatile anesthetic for the recipient can influence the incidence of PRS and graft outcomes [19,31]. However, in liver transplantation, the ischemic insult to the graft begins much earlier than the reperfusion phase—specifically with the clamping of the hepatic vessels during donor hepatectomy, followed by the period of cold storage. Consequently, effective preconditioning to prime the graft against IRI should ideally be initiated at the donor stage, prior to the onset of ischemia. Nevertheless, clinical evidence linking donor anesthetic management to recipient outcomes remains limited, with only a few studies in pediatric or deceased donor settings suggesting potential benefits of sevoflurane over propofol [9,10]. This scarcity is largely explained by the fact that LDLT has historically represented a minority of all liver transplants; for instance, in the United States, living donation accounted for only 5.7% of the 10,125 adult liver transplants performed in 2023 [23,32]. However, the annual volume of LDLT has increased steadily over the past decade, and recent evidence has established its superior patient survival and outcomes over deceased donor transplantation [33]. As the clinical importance of LDLT grows globally, identifying modifiable factors in donor management to further enhance these superior outcomes becomes increasingly essential.

The reduced incidence of PRS in the sevoflurane group may be attributed to the differing levels of ROS production induced by volatile agents. Previous experimental studies have demonstrated that desflurane induces significantly greater ROS production compared to sevoflurane in cardiomyocytes, which has been interpreted as a sign of potent preconditioning in healthy cells [34]. However, an acute surge of ROS upon reperfusion is a well-known driver of systemic oxidative stress and remote organ injury [24]. Consequently, the relatively higher ROS burden generated by desflurane could potentially exacerbate the profound vasodilation and hemodynamic instability characteristic of PRS. Since direct comparative studies between sevoflurane and desflurane in hepatocytes are currently lacking, further investigations are warranted to confirm these specific cellular mechanisms in the context of liver transplantation.

In our study, the sevoflurane group exhibited a significantly lower risk of prolonged ICU stay and mechanical ventilation. Previous research has shown that the severity of PRS significantly affects short-term outcomes for both patients and liver allografts [6]. Therefore, the reduced incidence of PRS observed in the sevoflurane group likely served as a mediator in preventing these adverse outcomes and facilitating a smoother postoperative course.

The effect of APC on donor sevoflurane is reflected in postoperative graft function markers. Ischemic hepatic injury typically leads to elevated AST and ALT levels due to hepatocellular necrosis and membrane leakage [35]. Furthermore, previous studies have reported higher peak AST levels in patients who developed PRS, suggesting that the hemodynamic instability associated with PRS may contribute to exacerbating hepatic injury [36]. In an experimental model, sevoflurane pretreatment significantly alleviated liver IRI, as evidenced by lower serum AST and ALT levels compared to the control group [37]. These findings are consistent with our results, which show improved laboratory profiles in the sevoflurane group, suggesting that sevoflurane may be more effective in preventing hepatic injury.

Patients with end-stage liver disease typically present with a state of chronic systemic inflammation, often driven by persistent immune activation and disrupted hepatic homeostasis [38,39]. Following liver transplantation, the restoration of graft function plays a pivotal role in resolving this inflammatory state [40]. Persistent postoperative inflammation often indicates delayed graft recovery and is associated with adverse outcomes [41,42]. In our study, the sevoflurane group exhibited significantly faster recovery trends in systemic inflammatory indices, specifically CAR and NLR, compared to the desflurane group. These findings suggest that the preservation of liver function, facilitated by donor sevoflurane preconditioning, may have contributed to the accelerated resolution of systemic inflammation. Consequently, prioritizing sevoflurane for donor anesthesia may serve as a strategic intervention to optimize the recipient’s physiological reserve and enhance early postoperative recovery.

This study has several limitations. First, the retrospective design inherently introduces the potential for selection bias and unmeasured confounding factors. Specifically, the choice of volatile anesthetic agent (sevoflurane versus desflurane) for the living donor was determined at the discretion of the attending anesthesiologist, meaning that selection bias regarding anesthetic administration cannot be entirely excluded. To address these methodological limitations and enhance the validity of our findings, we employed a rigorous propensity score matching analysis. Second, we did not analyze the exact concentration of donor anesthetic exposure, which could influence the magnitude of the preconditioning effect, although the anesthetic dose was titrated to maintain a target anesthetic depth monitored by electroencephalography-based indices. Future studies incorporating detailed pharmacokinetic data are warranted. Finally, although a large cohort was used to ensure statistical power, the population was recruited from a single center, which may limit the generalizability of our findings and necessitate multi-center validation.

## 4. Materials and Methods

### 4.1. Patients

This retrospective analysis examined a cohort of patients who underwent LDLT at Asan Medical Center (Seoul, Republic of Korea) from January 2008 to December 2025. The study population comprised adult patients (aged ≥ 18 years) who received LDLT. Patients were excluded based on the following criteria: (1) preoperative renal impairment, defined as a diagnosis of chronic kidney disease, hepatorenal syndrome, or a serum creatinine level > 1.5 mg/dL; and (2) missing data regarding postoperative outcomes. After applying these selection criteria, a total of 5006 adult LDLT recipients were included in the study.

### 4.2. Anesthetic Management and Surgical Procedures

The choice of volatile anesthetic agent for living donors was determined by the attending anesthesiologist. Donors were induced with propofol and maintained with either sevoflurane or desflurane, along with remifentanil and rocuronium. The concentration of the volatile anesthetic was adjusted to maintain a Bispectral Index (BIS) or SedLine value below 50.

Perioperative anesthetic management for recipients was conducted according to the protocol described in our previous studies [43,44,45]. Anesthesia was induced with propofol, fentanyl, and rocuronium and maintained with volatile anesthetics, along with continuous infusions of fentanyl and rocuronium. Standard monitoring included invasive arterial blood pressure measurement via the radial and femoral arteries, as well as pulmonary artery catheterization for hemodynamic assessment. Vasopressors, including norepinephrine, were administered as needed to maintain a MAP ≥ 65 mmHg.

The native liver was dissected to expose the inferior vena cava. Subsequently, the donor graft was implanted via hepatic vein anastomosis, followed by end-to-end portal vein anastomosis. Graft reperfusion was initiated after completing the portal vein anastomosis. During this reperfusion phase, hemodynamic changes were closely monitored to detect post-reperfusion syndrome, which was managed with bolus doses of vasopressors, calcium gluconate, or sodium bicarbonate to stabilize hemodynamics and correct associated electrolyte or acid-base imbalances. This was followed by sequential anastomosis of the hepatic artery and bile duct. Detailed surgical procedures for LDLT have been described in previous studies [43,44].

### 4.3. Clinical Data and Outcomes

Comprehensive clinical data of donors and recipients were extracted from the electronic medical record system of Asan Medical Center. Recipient demographic variables included age, sex, body mass index, and comorbidities such as diabetes mellitus, hypertension, and cardiovascular disease history (including coronary artery disease, congestive heart failure, or cerebrovascular accident). Preoperative clinical data encompassed the etiology of cirrhosis, the presence of hepatocellular carcinoma, transplant era (2008–2012, 2013–2017, 2018–2022, and after 2023), and severity scores, including the Model for End-Stage Liver Disease-Sodium (MELD-Na) and Child-Turcotte-Pugh scores. Donor and graft-related variables, such as donor age, donor sex, graft fatty change, and graft-to-recipient weight ratio, were also recorded. Intraoperative details included the incidence of PRS, anesthesia time, ischemic time, incidence of massive transfusion (defined as ≥10 units of packed red blood cells within 24 h or ≥4 units within 1 h), and intraoperative norepinephrine use [46]. Additionally, data on early allograft dysfunction (EAD) were collected, defined by the presence of one or more of the following criteria: TB ≥ 10 mg/dL, INR ≥ 1.6 on postoperative day 7, and peak AST or ALT levels ≥ 2000 U/L within the first 7 postoperative days [47]. Data regarding the median length of ICU stay and mechanical ventilation, as well as prolonged ICU stay (>30 days), prolonged mechanical ventilation (>14 days), and 1-year graft failure rates were also recorded. Furthermore, postoperative laboratory values—including TB, INR, AST, and ALT—along with inflammatory markers such as the CAR, NLR and PLR, were also collected.

The primary outcome of this study was to evaluate the impact of donor sevoflurane versus desflurane anesthesia on the incidence of PRS in recipients. Secondary outcomes included the incidence of EAD, median duration of ICU stay and mechanical ventilation, prolonged ICU stay (>30 days), prolonged mechanical ventilation (>14 days), 1-year graft failure, postoperative laboratory values, and postoperative inflammatory markers.

### 4.4. Statistical Analysis

Categorical variables are presented as frequencies and percentages, while continuous variables are expressed as means ± standard deviations (SD) or medians with interquartile ranges. Group comparisons were conducted using Student’s *t*-test or Mann–Whitney U test for continuous variables, and the Chi-square test or Fisher’s exact test for categorical variables, as appropriate.

To balance baseline characteristics between the two groups (Sevoflurane vs. Desflurane), PSM was performed. PSM was conducted using a 1:1 nearest-neighbor algorithm and was calculated using a logistic regression model incorporating relevant demographic, preoperative, intraoperative variables, and transplant era. The balance of covariates was assessed using the standardized mean difference (SMD), with an SMD < 0.1 considered indicative of a negligible imbalance.

The associations between donor anesthetic type and binary clinical outcomes were evaluated across three models: a crude unadjusted analysis, a multivariable-adjusted logistic regression using backward stepwise elimination, and an analysis within the propensity score-matched cohort. Results are reported as OR with 95% CI. To evaluate postoperative trends of continuous laboratory variables and inflammatory markers over time (postoperative days 1–7), linear quantile mixed models were applied. A group-by-time interaction was assessed to determine whether the longitudinal trends of these variables differed significantly between groups. All statistical analyses were conducted using R software (version 4.5.1; R Foundation for Statistical Computing, Vienna, Austria).

### 4.5. Ethical Statements

Approval of this study was obtained from the Institutional Review Board of our center (protocol number: 2026-0247). Due to the retrospective design, the board waived the requirement to obtain informed consent from the participants.

## 5. Conclusions

Donor sevoflurane anesthesia was associated with a significantly lower risk of PRS and improved early postoperative recovery in LDLT recipients compared to desflurane. These findings suggest that the choice of volatile anesthetics for living donors is a potentially modifiable factor that can optimize recipient outcomes. Given the comparable safety profile for donors, sevoflurane may be considered the preferred agent for donor anesthesia in LDLT to provide better intraoperative hemodynamic stability and enhance the quality of recovery in recipients.

## Figures and Tables

**Table 1:** Baseline characteristics of the study population.

	Before PSM (*n* = 5006)				After PSM (*n* = 1882)			
	Sevoflurane (*n* = 2123)	Desflurane (*n* = 2883)	SMD	*p*	Sevoflurane (*n* = 941)	Desflurane (*n* = 941)	SMD	*p*
Demographic variables								
Age (years)	52.7 ± 8.6	55.4 ± 9.4	0.310	<0.001	53.9 ± 8.5	54.4 ± 8.9	0.062	0.178
Sex, male	1582 (74.5)	2008 (69.6)	0.109	<0.001	700 (74.4)	678 (72.1)	0.053	0.274
Body mass index (kg/m^2^)	24.3 ± 3.4	24.2 ± 3.7	0.020	0.477	24.3 ± 3.5	24.4 ± 3.7	0.034	0.460
Diabetes mellitus	484 (22.8)	796 (27.6)	0.111	<0.001	240 (25.5)	244 (25.9)	0.010	0.874
Hypertension	316 (14.9)	633 (22.0)	0.183	<0.001	163 (17.3)	181 (19.2)	0.050	0.311
Cardiovascular disease	333 (15.7)	373 (12.9)	0.079	0.007	149 (15.8)	166 (17.6)	0.048	0.323
Transplant Era (%)			1.690	<0.001			<0.001	1.000
2008–2012	1109 (52.2)	134 (4.6)			133 (14.1)	133 (14.1)		
2013–2017	749 (35.3)	711 (24.7)			545 (57.9)	545 (57.9)		
2018–2022	224 (10.6)	1347 (46.7)			223 (23.7)	223 (23.7)		
After 2023	41 (1.9)	691 (24.0)			40 (4.3)	40 (4.3)		
Etiology and lab results								
Hepatocellular carcinoma	1095 (51.6)	1490 (51.7)	0.002	0.965	485 (51.5)	484 (51.4)	0.002	1.000
Viral hepatitis	1532 (72.2)	1671 (58.0)	0.301	<0.001	625 (66.4)	593 (63.0)	0.071	0.135
Alcoholic liver disease	361 (17.0)	762 (26.4)	0.230	<0.001	216 (23.0)	197 (20.9)	0.049	0.316
Creatinine (mg/dL)	0.8 ± 0.2	0.8 ± 0.2	0.086	0.003	0.8 ± 0.2	0.8 ± 0.2	0.089	0.054
MELD-Na score	15.0 ± 7.7	13.6 ± 6.7	0.200	<0.001	14.3 ± 7.3	14.1 ± 7.1	0.020	0.672
MELD-Na score ≥ 20	526 (24.8)	530 (18.4)	0.156	<0.001	212 (22.5)	189 (20.1)	0.060	0.216
CTP score	8.0 ± 2.3	7.7 ± 2.2	0.101	<0.001	7.9 ± 2.2	7.8 ± 2.2	0.054	0.243
Donor and graft variables								
Donor age (years)	28.2 ± 8.5	31.0 ± 9.1	0.322	<0.001	28.7 ± 8.7	29.4 ± 8.7	0.090	0.050
Donor sex, male	1476 (69.5)	1809 (62.7)	0.144	<0.001	632 (67.2)	624 (66.3)	0.018	0.732
Total fatty change (%)	3.9 ± 6.1	4.1 ± 6.4	0.038	0.182	4.2 ± 6.4	4.2 ± 7.2	<0.001	0.992
Low GRWR (<0.8)	110 (5.2)	247 (8.6)	0.134	<0.001	67 (7.1)	67 (7.1)	<0.001	1.000
Intraoperative variables								
Anesthesia time (h)	14.4 ± 2.5	13.2 ± 2.3	0.521	<0.001	13.8 ± 2.3	13.7 ± 2.5	0.055	0.232
Massive transfusion	773 (36.4)	955 (33.1)	0.069	0.017	317 (33.7)	310 (32.9)	0.016	0.769
Cold ischemic time (min)	84.5 ± 30.7	83.0 ± 26.2	0.053	0.062	83.4 ± 23.2	83.0 ± 27.2	0.019	0.683
Warm ischemic time (min)	43.2 ± 15.5	38.8 ± 15.0	0.293	<0.001	41.7 ± 14.8	41.6 ± 15.6	0.006	0.905
Total ischemic time (min)	127.7 ± 35.6	121.8 ± 32.3	0.175	<0.001	125.1 ± 28.5	124.6 ± 33.9	0.018	0.698
Norepinephrine use	1133 (53.4)	2721 (94.4)	1.056	<0.001	791 (84.1)	799 (84.9)	0.023	0.656
Outcomes								
PRS	1093 (51.5)	2319 (80.4)		<0.001	602 (64.0)	676 (71.8)		<0.001
EAD	205 (9.7)	220 (7.6)		0.013	67 (7.1)	77 (8.2)		0.435
ICU stay (day)	3.0 [2.0–5.0]	3.0 [2.0–8.0]		0.630	3.0 [2.0–5.0]	3.0 [2.0–5.0]		0.039
MV (day)	2.0 [2.0–3.0]	2.0 [2.0–3.0]		0.084	2.0 [2.0–3.0]	2.0 [2.0–3.0]		0.880
Prolonged ICU stay	30 (1.4)	100 (3.5)		<0.001	12 (1.3%)	26 (2.8%)		0.032
Prolonged MV	47 (2.2)	133 (4.6)		<0.001	24 (2.6%)	37 (3.9%)		0.120
1-year graft failure	131 (6.2)	120 (4.2)		0.002	48 (5.1%)	40 (4.3%)		0.445

Values are expressed as mean ± standard deviation, median [interquartile range], or number of patients (%) as appropriate. Abbreviations: PSM, propensity score matching; CTP score, Child-Turcotte-Pugh score; MELD-Na score, Model for End-Stage Liver Disease sodium score; GRWR, graft-to-recipient weight ratio; INR, international normalized ratio; PRS, post-reperfusion syndrome; EAD, early allograft dysfunction; ICU, intensive care unit; MV, mechanical ventilation; SMD, standardized mean difference.

**Table 2:** Multivariate logistic regression analysis for post-reperfusion syndrome.

	Univariate Analysis			Multivariate Analysis		
	OR	95% CI	*p*	OR	95% CI	*p*
Anesthetic type (Sevoflurane)	0.26	0.23–0.29	<0.001	0.47	0.41–0.55	<0.001
Age (years)	1.04	1.03–1.04	<0.001	1.03	1.02–1.03	<0.001
Sex, male	0.89	0.78–1.01	0.081			
Diabetes mellitus	1.44	1.25–1.66	<0.001	1.21	1.04–1.42	0.016
Hypertension	1.32	1.13–1.55	<0.001			
Cardiovascular disease	0.96	0.81–1.14	0.651	0.86	0.71–1.04	0.128
Hepatocellular carcinoma	0.86	0.76–0.97	0.013			
Viral hepatitis	0.56	0.49–0.64	<0.001			
Alcoholic liver disease	1.98	1.70–2.32	<0.001	1.35	1.13–1.61	<0.001
MELD-Na score ≥ 20	1.04	0.90–1.20	0.642			
CTP score	1.05	1.02–1.08	<0.001	1.06	1.02–1.09	<0.001
Donor age (years)	1.02	1.01–1.03	<0.001			
Donor sex, male	0.97	0.85–1.10	0.609			
Total fatty change (%)	1.02	1.01–1.03	<0.001	1.01	1.00–1.02	0.119
Low GRWR (<0.8)	0.83	0.67–1.05	0.117	0.64	0.50–0.82	<0.001
Anesthesia time (h)	0.87	0.85–0.90	<0.001	0.93	0.90–0.97	<0.001
Massive transfusion	1.12	0.99–1.27	0.082	1.25	1.06–1.48	0.008
Total ischemic time (min)	1.0	0.99–1.00	<0.001	1.00	1.00–1.00	0.006
Norepinephrine use	5.62	4.89–6.48	<0.001	3.40	2.89–4.00	<0.001

Abbreviations: OR, odds ratio; CI, confidence interval; MELD-Na, Model for End-Stage Liver Disease sodium score; CTP score, Child-Turcotte-Pugh score; GRWR, graft-to-recipient weight ratio.

**Table 3:** Impact of Donor Anesthetic Type on Post-Reperfusion Syndrome and Early Recovery.

		Crude			Multivariable Adjusted *		Propensity Score-Matched		
		Event/n	OR (95% CI)	*p*-Value	OR (95% CI)	*p*-Value	Event/n	OR (95% CI)	*p*-Value
Post-reperfusion syndrome	Sevoflurane	1093/2123	0.26 (0.23–0.29)	<0.001	0.47 (0.41–0.55)	<0.001	602/941	0.70 (0.57–0.85)	<0.001
	Desflurane	2319/2883	1		1		676/941	1	
Prolonged ICU stay	Sevoflurane	30/2123	0.40 (0.26–0.59)	<0.001	0.49 (0.30–0.79)	0.004	12/941	0.45 (0.22–0.89)	0.025
	Desflurane	100/2883	1		1		26/941	1	
Prolonged MV	Sevoflurane	47/2123	0.47 (0.33–0.65)	<0.001	0.60 (0.41–0.88)	0.011	24/941	0.64 (0.37–1.07)	0.093
	Desflurane	133/2883	1		1		37/941	1	

Data are presented as incidence (event/n) and odds ratios. * Adjusted for the identical set of covariates included in the multivariable analysis presented in Table 2. Abbreviations: OR, odds ratio; CI, confidence interval; ICU, intensive care unit; MV, mechanical ventilation.

**Table 4:** Comparison of maximum postoperative laboratory values and inflammatory markers between groups.

	Sevoflurane	Desflurane	*p*
Total bilirubin (mg/dL)	5.2 [3.5–8.0]	5.1 [3.5–7.7]	0.240
INR	1.95 [1.76–2.21]	1.99 [1.81–2.27]	0.001
AST (IU/L)	272 [193–399]	289 [201–442]	0.018
ALT (IU/L)	259 [174–378]	264 [186–414]	0.035

Data are expressed as median [interquartile range]. Abbreviations: INR, international normalized ratio; AST, aspartate aminotransferase; ALT, alanine aminotransferase.

## Data Availability

The datasets used and/or analyzed during the current study are available from the corresponding author upon reasonable request.

## Abbreviations

The following abbreviations are used in this manuscript:

ALT	Alanine Aminotransferase
APC	Anesthetic Preconditioning
AST	Aspartate Aminotransferase
CAR	C-reactive Protein-to-Albumin Ratio
CI	Confidence Interval
CTP	Child-Turcotte-Pugh
EAD	Early Allograft Dysfunction
GRWR	Graft-to-Recipient Weight Ratio
ICU	Intensive Care Unit
INR	International Normalized Ratio
LDLT	Living Donor Liver Transplantation
LT	Liver Transplantation
MAP	Mean Arterial Pressure
MELD-Na	Model for End-Stage Liver Disease-Sodium
MV	Mechanical Ventilation
NLR	Neutrophil-to-Lymphocyte Ratio
OR	Odds Ratio
PLR	Platelet-to-Lymphocyte Ratio
PRS	Post-Reperfusion Syndrome
PSM	Propensity Score Matching
ROS	Reactive Oxygen Species
SD	Standard Deviation
SMD	Standardized Mean Difference
TB	Total Bilirubin
